# Incision of the Elbow Joint to Relieve Anchylosis

**Published:** 1871-10

**Authors:** 


Ixcision of Elbow Joint to Relieve Anchylosis.—Geo. E. Fenwick reports a very interesting case of partial anchylosis of the elbow joint, in the straight position the result of an unreduced dislocation, upon which he operated by excision with most excellent results. The case when first treated was mistaken by the attending surgeon for fracture, and was bandaged up in a straight position, and kept so for forty days. When she first came under Dr. Fenwick's care, reduction was impossible. Extension, flexion, supination and pronation are now nearly perfect.—*Canada Medical Journal.*

